# Early warming stress on rainbow trout juveniles impairs male reproduction but contrastingly elicits intergenerational thermotolerance

**DOI:** 10.1038/s41598-021-96514-1

**Published:** 2021-08-23

**Authors:** Arno Juliano Butzge, Tulio Teruo Yoshinaga, Omar David Moreno Acosta, Juan Ignacio Fernandino, Eduardo Antônio Sanches, Yara Aiko Tabata, Claudio de Oliveira, Neuza Sumico Takahashi, Ricardo Shohei Hattori

**Affiliations:** 1 Department of Structural and Functional Biology, Institute of Biosciences, Botucatu São Paulo State University (UNESP), Botucatu, 18618-689 Brazil; 2 Department of Surgery, School of Veterinary Medicine and Animal Sciences, University of São Paulo, São Paulo, 05508-270 Brazil; 3 Laboratorio de Biología del Desarrollo, Instituto Tecnológico de Chascomús (INTECH), Consejo Nacional de Investigaciones Científicas y Técnicas/Universidad Nacional de San Martín (CONICET/UNSAM), 7130 Chascomús, Argentina; 4 Fishery Engineering Course and Aquaculture Centre (CAUNESP), São Paulo State University, Registro, 11900-000 Brazil; 5 Salmonid Experimental Station At Campos Do Jordão, UPD-CJ (APTA/SAA), Campos do Jordão, 12460-000 Brazil; 6 Centro de Pesquisa de Aquicultura, Sao Paulo Fisheries Institute (APTA/SAA), São Paulo, 05001-900 Brazil

**Keywords:** Animal physiology, Reproductive biology

## Abstract

The exposure of adult fish to warm or high temperatures is known to impair reproduction, yet the long-term reproductive impacts for treatments at early life are not well clarified. This study aimed to evaluate the effects of warm temperature (WT) during juvenile stage on gonad maturation, gamete quality, and offspring thermotolerance in rainbow trout. While the comparison of basic reproductive parameters in WT females did not reveal any kind of impairment, many WT males showed an atrophied, undeveloped gonad, or a smaller testis with lower milt volume; sperm quality parameters in WT males and deformity rates in the respective progeny were also highly affected. However, despite of such negative effects, many of the remaining progeny presented better rates of survival and growth when exposed to the same conditions as those of parental fish (WT), suggesting that thermal stress in *parr* stage males elicited intergenerational thermotolerance after a single generation. The present results support that prolonged warming stress during early life stages can adversely affect key reproductive aspects, but contrastingly increase offspring performance at upper thermal ranges. These findings have implications on the capacity of fish to adapt and to cope with global warming.

Water temperature comprises an important modulatory factor with critical roles on fish reproduction^1^. During early life stages, the destiny of gonadal sex differentiation in gonochoristic species can be irreversibly driven towards either female or male by temperature, overcoming the predisposed sex determined by genotypic factors^2–5^. The appearance of sex-reversed fish and the concomitant skews in sex ratios have great implications from ecological perspectives due to their impacts on population structure^6^.

Another effect of temperature on reproduction occurs through regulation of the reproductive cycle, either by promoting^7,8^ or suppressing gametogenesis^9^. However, chronic exposure at those temperatures or acute thermal stress at even higher temperatures can cause opposite inhibitory effects on spermatogenesis^10,11^. In ovaries, although warm conditions are also able to hasten gametogenesis as in males^7^, high temperatures that do show clear inhibitory effects on spermatogenesis do not necessarily induce comparable changes in oocyte development^12^. At sub-lethal, high temperature conditions, the survival of testicular somatic-supporting cells as well as the germ cells can be severely affected, whereby undifferentiated spermatogonia seems to be more tolerant to depletion by apoptosis than the differentiated ones, such as spermatocytes, spermatids, and spermatozoa^10,11^. The mechanism of heat-induced germ cell depletion is not well understood, but the Sertoli cells are likely involved, since apoptosis in these cells has been detected along with germ cells^10,11^. On the other hand, undifferentiated oogonia seem to be more susceptible than the differentiated oocytes upon exposure to those temperatures^12^, suggesting that high temperatures affect fish reproduction in a sex-specific manner^13^.

Although the effects of thermal stress on fish reproduction have been assessed in some species, the implications on their reproductive capacity have not been well explored, especially in terms of gamete quality and offspring performance. Furthermore, the performance of offspring produced by fish exposed to warm water temperature has not been well evaluated yet. Research about how temperature acts on the fish germ line, on gametes production or quality, and on progeny fitness might provide important insights for the evaluation of environmental changes (e.g., global warming) on wild populations and extensive aquaculture^14^. In this regard, salmonids are an excellent group of fish to evaluate the effects of increasing temperature because they include several cold-water species that are born in freshwater environments and then migrate downward to the river mouths until reaching the sea. Also, some species present variants that spend their entire life cycle in inland waters (landlocked) such as the rainbow trout (*Oncorhynchus mykiss*) and the Atlantic salmon (*Salmo salar*). But regardless of these different life cycles, many salmonids have a high likelihood to experience warm temperatures and hypoxic conditions during the juvenile stage^15,16^. Some of those effects include impairment of steroidogenesis and vitellogenin synthesis^17^, and advance or delay in oocyte maturation in females^18,19^. In the case of males, impairment of spermatogenesis and reduced milt volume are reported in fish exposed to high temperature^20,21^.

In this study, we used the rainbow trout (*Oncorhynchus mykiss*) as an experimental model to evaluate the effects of prolonged treatment at warm temperatures during juvenile stage on several reproduction parameters in female and male adults (see the experimental design in Fig. 1). We also compared survival and growth performances of the respective progeny under warm temperature in juveniles and the upper thermal tolerance in adults in order to investigate the intergenerational inheritance of thermotolerance.

**Figure 1:**
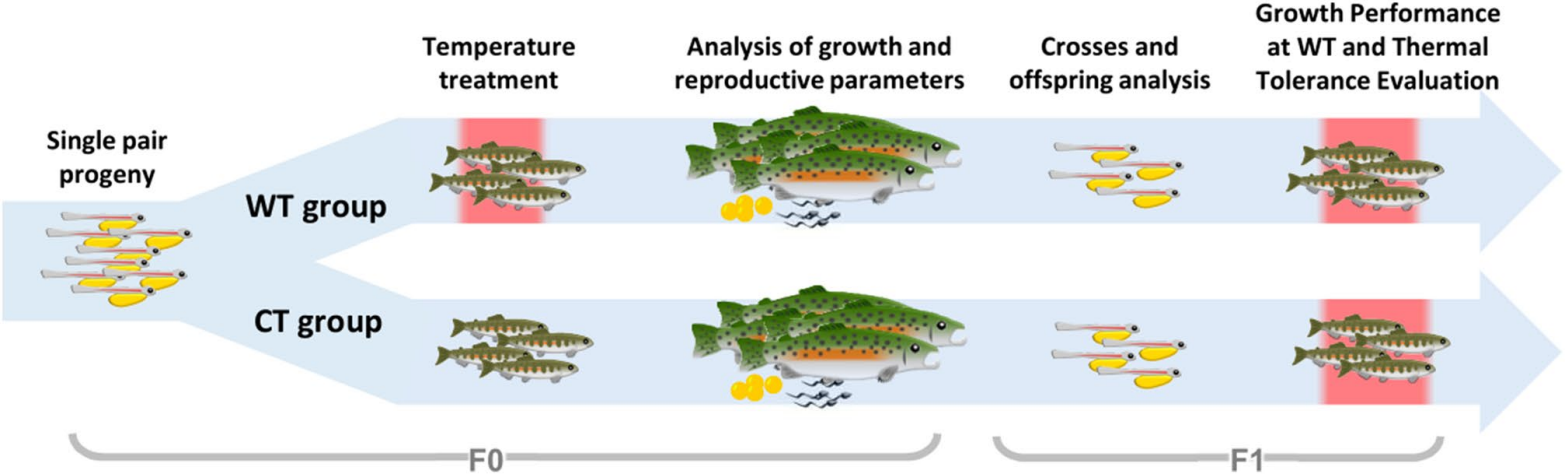
Overview representation of the experimental design. Rainbow trout juveniles obtained from a single pair cross were divided in two groups. At six-months, one group was exposed to warm temperature (WT) for three months in a local fish farm (Pindamonhangaba) while control group was maintained at Salmonid Experimental Station (Campos do Jordão). After this period, the treated group returned to control temperature until reaching sexual maturity for the analysis of growth and reproductive parameters. Finally, survival and growth performance of respective progeny at WT and upper thermal tolerance were evaluated.

## Results

### Effects of warm temperature on body growth and gonads of juveniles

**Figure 2:**
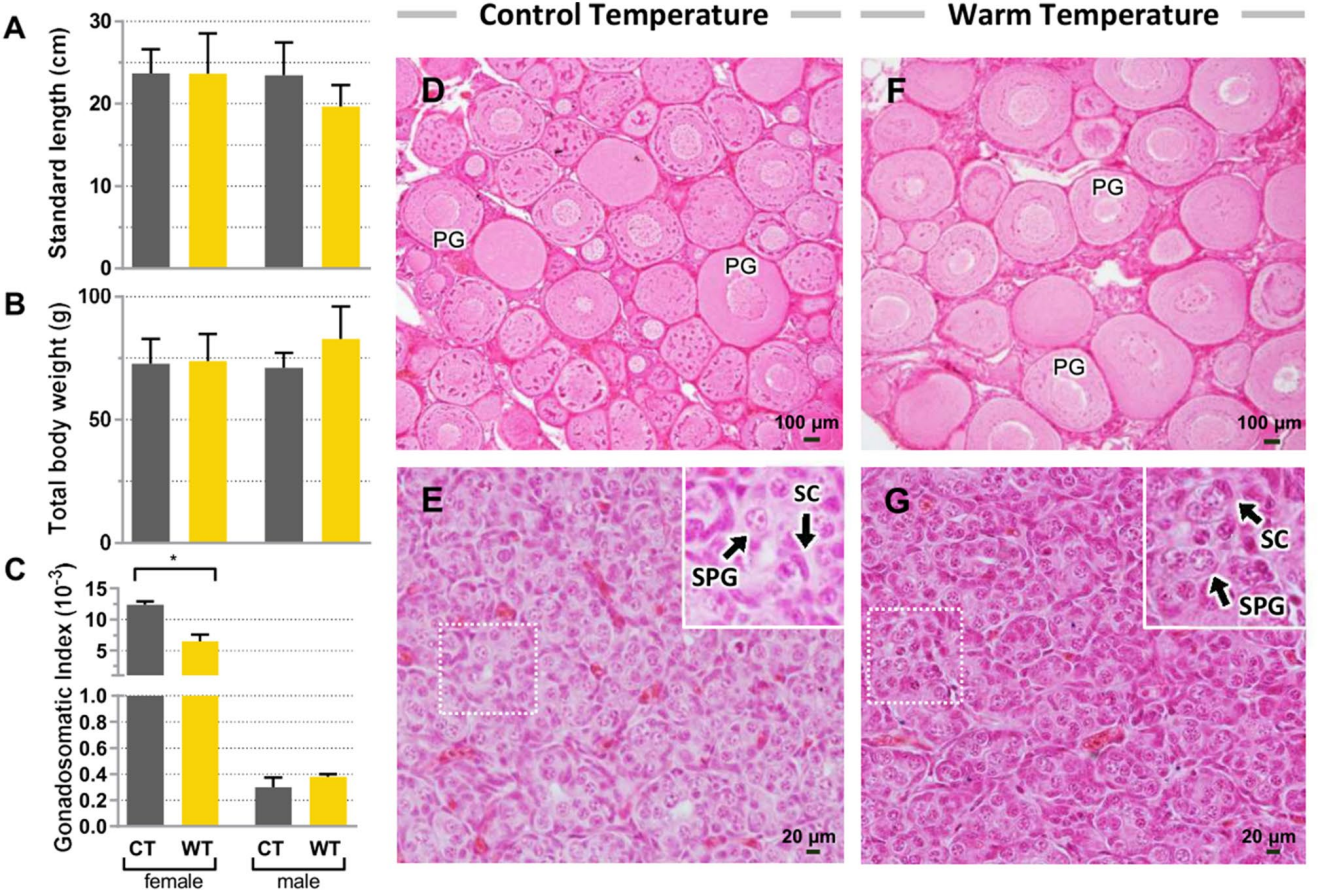
Growth and reproductive parameters of F0 juveniles from control (CT) and warm temperature (WT) groups. (**A**) Total body weight, (**B**) standard length, and (**C**) gonadosomatic index in females and males after thermal treatment. (**D**, **F**) Ovary and (**E**, **G**) testis histology from F0 juvenile fish three months after thermal treatment. The enlarged images in E and G refer to the respective dotted boxes. PG: primary growth oocytes; SPG: spermatogonia; CS: Sertoli cells. Histograms are presented as mean ± S.E.M. Asterisks (*) indicate significant difference for *p* < 0.05. Data were analyzed in GraphPad Prism (version 5.02 for Windows, GraphPad Software, La Jolla California USA, www.graphpad.com).

At the end of the experiment (3 months) with F0 juvenile fish, the warm temperature group (WT) showed lower survival rate than control group (CT) (70% and 97%, respectively), but growth parameters such as standard length (mean ± SD = 21.67 ± 9.41 cm and 23.57 ± 8.76 cm, respectively), and body weight (81.61 ± 28.79 g and 71.73 ± 18.26 g, respectively) did not differ significantly (Fig. 2A, B). The gonadosomatic index in females was lower in WT compared to CT (0.0007 ± 0.0003 vs 0.0012; *p* = 0.002) whereas no difference was found for males (Fig. 2C). Histological analyses of ovaries (Fig. 2D and F) and testis (Fig. 2E and G) did not reveal clear differences between WT and CT groups.

### Reproductive parameters of adults

Females from WT group were significantly smaller than those from CT group (*p* = 0.01) and presented higher condition factor (K) (*p* = 0.03). Nevertheless, no differences were found in body weight (Supplementary Fig. 1). Fecundity rates, oocyte mean weight, and the percentage of non-ovulated females did not differ between females of WT and CT group (Supplementary Fig. 2; Table 1).

**Table 1:** Number of mature and immature adult fish at both control (CT) and warm temperature groups 16 mo after treatment.

Maturity stage	Females		Males	
	WT	CT	WT*	CT
Matures	6 (75%)	13 (81.25%)	12 (60%)	17 (100%)
Immatures	2 (25%)	3 (18.75%)	8 (40%)	0 (0%)

Number in parentheses represent the frequency of animals in each group.

*Represent significant difference for Fisherʼs test considering *p* < 0.05.

**Figure 3:**
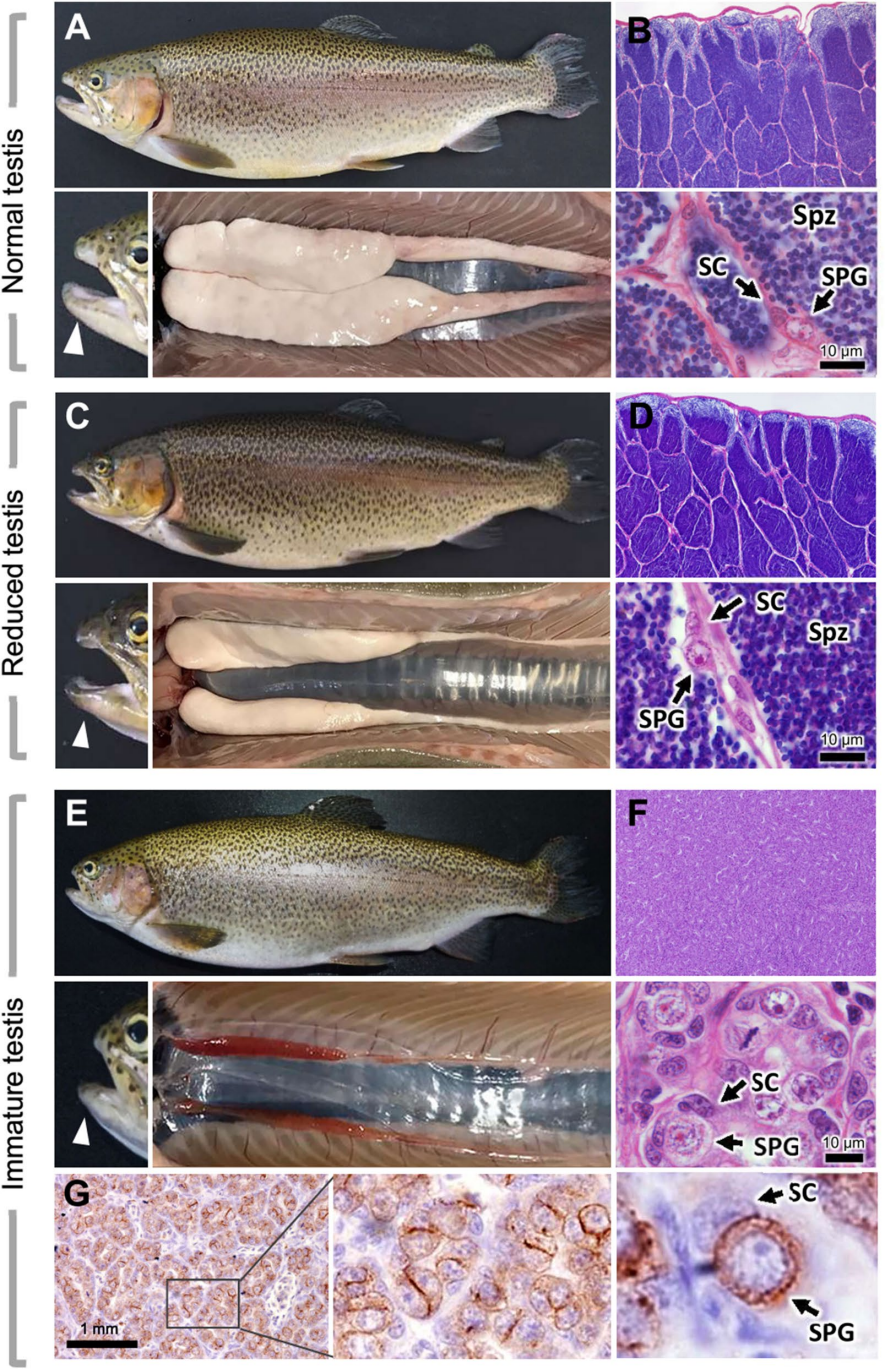
External body appearance and morpho-histological analysis of gonads in warm and control temperature males. (**A**, **B**) Adult trout with typical male phenotype presenting a hook-like jaw, a large whitish testis with abundant spermatozoa; spermatogonia and Sertoli cells are also present. (**C**, **D**) Adult trout with typical male phenotype as in (**A**), but with a smaller testis; testis histology presents similar aspect to (**B**). (**E**, **F**) Adult trout without male-specific secondary sexual characteristics, presenting a thin, reddish gonad, without spermatozoa; most germ cells are spermatogonia. SPG: spermatogonia; CS: Sertoli cells; Spz: spermatozoa. Arrowheads indicate the jaw, which has a hook-like morphology in A and C (mature males), but not in E (immature male). (**G**) Immunohistochemical detection of undifferentiated spermatogonia in immature testis with a cell surface marker.

WT males were significant smaller (*p* = 0.001) and had lower body weight than control males (*p* = 0.007); hence, condition factor was significantly higher in WT group (*p* = 0.002) (Supplementary Fig. 1), as in females. Gonad dissection and histological analyses in some of those fish revealed three patterns of testis morphology. The first one consisted in a large whitish testis, similar to those of CT males (Fig. 3A). In the second pattern, a smaller whitish testis was detected and correlated with males with low relative milt volume (Fig. 3C). The third pattern was found for immature males and consisted in a thinner gonad with a reddish color (Fig. 3E). Histological analyses revealed the presence of some undifferentiated spermatogonia and a high quantity of spermatozoa in the first two patterns (Fig. 3B and D) whereas the third pattern was characterized by undifferentiated spermatogonia without any spermatozoa or spermatocytes (Fig. 3F), as revealed by immunohistochemistry analysis with an antibody for undifferentiated spermatogonia (Fig. 3G), which resembled the immature testis of F0 juveniles (Fig. 2E).

**Figure 4:**
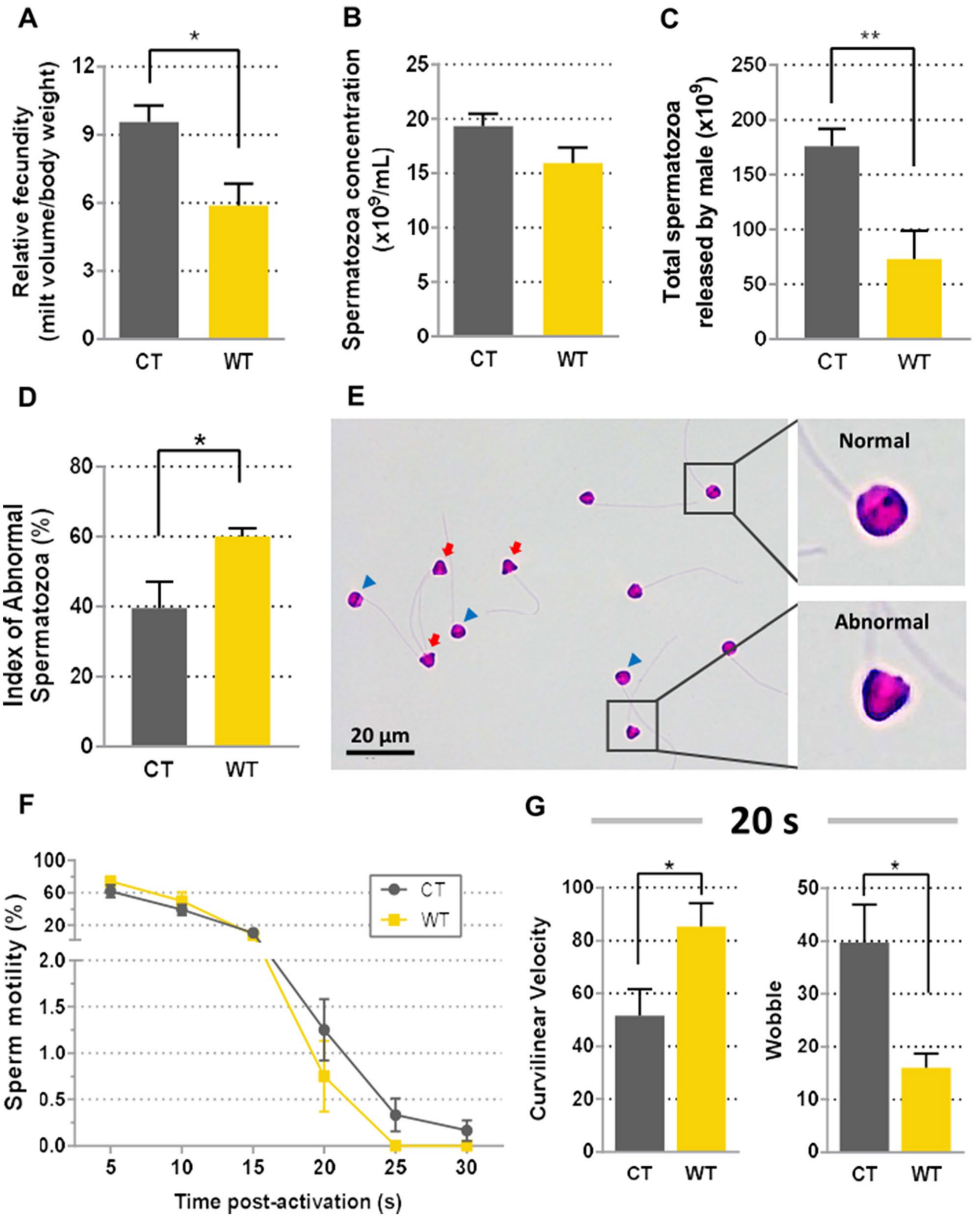
Fecundity of males and sperm motility parameters in control (CT) and warm (WT) temperature groups. (**A**) The relative fecundity was significantly lower in WT than in CT group, (**B**) but without differences in sperm concentration, (**C**) the total number of sperm released per male was significantly lower in the WT than in CT. (**D**, **E**) Morphological comparison between the percentages of spermatozoa with normal-ovoid and abnormal-triangular heads. (**F**) Sperm motility analyses in CT and WT males between 5 and 30 s post-activation. (**G**) Curvilinear velocity and wobble showed opposite patterns at 20 s. Histograms are presented as mean ± S.E.M. Asterisks indicate significant difference for *p* < 0.05. Data were analyzed in GraphPad Prism (version 5.02 for Windows, GraphPad Software, La Jolla California USA, www.graphpad.com).

The relative milt volume was also lower in WT than CT group (*p* = 0.003) (Fig. 4A), with no difference in the concentration of spermatozoa (Fig. 4B). The estimated total amount of spermatozoa was reduced by about 57% in WT males (*p* = 0.001) (Fig. 4C). A proportion of males did not show secondary sexual characters and did not release any milt. These males were classified as immature males and they were not detected in any of CT males (Table 1). The morphological analysis of the sperm showed a 1.5-fold higher the percentage of abnormal cells in WT compared to CT group (59.99% and 39.56%, respectively; *p* = 0.03) (Fig. 4D and E).

Computer analysis of sperm motility showed no differences for all parameters among 5 and 15 s, but at 20 s curvilinear velocity was higher (*p* = 0.046) while wobble was lower in WT compared to CT males (*p* = 0.032) (Fig. 4G). At both 25 and 30 s, sperm motility was detected in 66.67% and 33.33% of CT males, respectively (Fig. 4F), whereas in WT group, no male showed sperm motility in any of these time points.

### Fertilization, hatching, and abnormality rates in offspring

**Figure 5:**
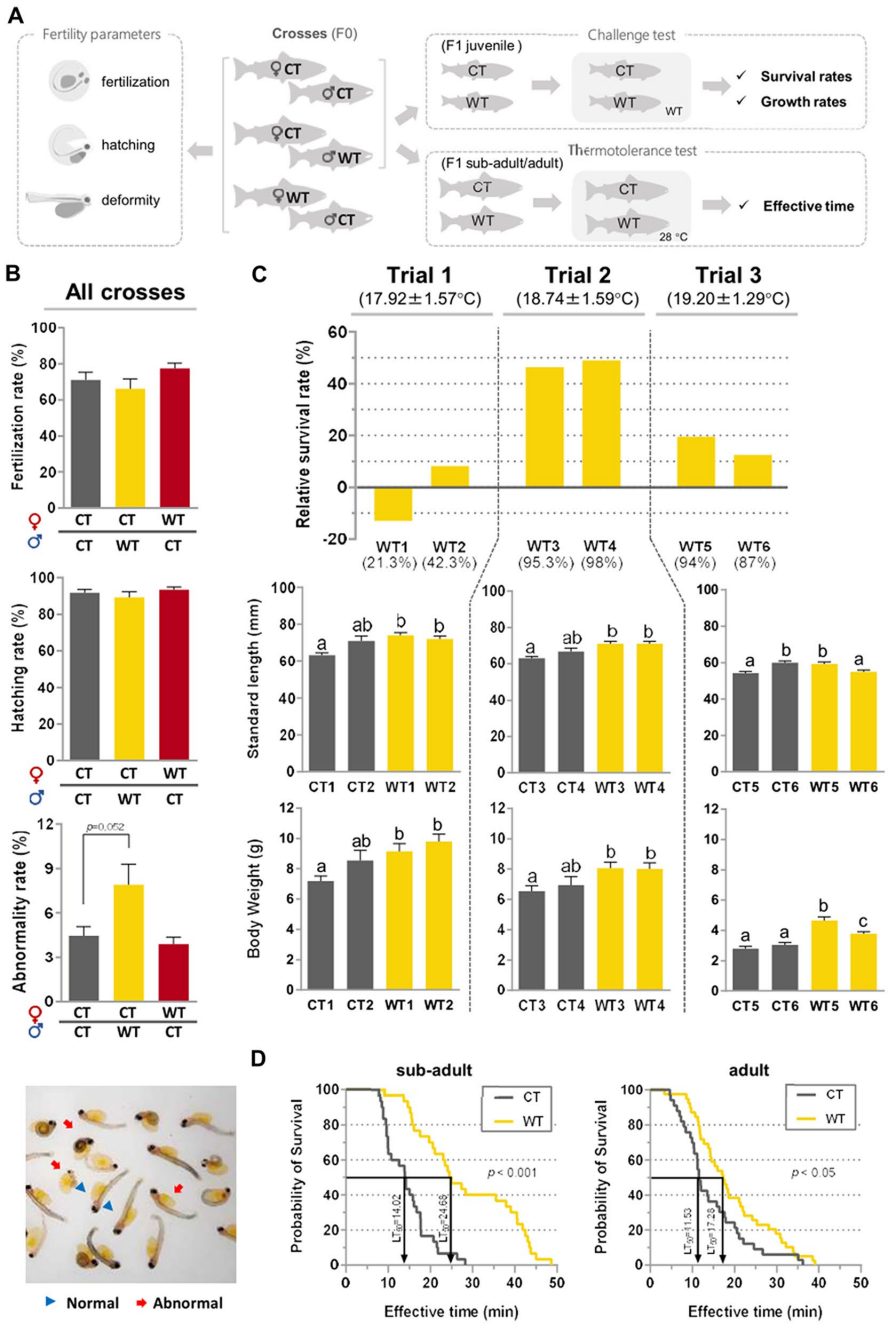
Performance of progeny derived from WT and CT males. (**A**) Schematic representation the experimental design and analysis performed with the progeny. (**B**) Fertilization, hatching, and abnormality rates of progeny produced by gametes from control (CT) and warm temperature (WT) groups. Rates of fertilization, hatching, and abnormality did not differ among the crosses. Crosses between CT females X WT males showed higher abnormality rates, with typical body deformities shown in the image just below. (**C**) Normalized survival rate (in relation to the average of respective CT groups) and growth parameters in F1 progeny derived from the CT/WT males submitted to warm temperature. Temperature values below each trial represent the average temperature ± SD during challenge experiment. Numbers between brackets in the graph of survival represent the absolute survival rates for each group. (**D**) Comparative analysis of upper thermal tolerance using F1 sub-adults and adults from CT and WT males using effective time (ET). LT_50_ (median lethal temperature) values are indicated for each curve. Histograms are presented as mean ± S.E.M. Different letters indicate significant difference for *p* < 0.05. Data were analyzed in GraphPad Prism (version 5.02 for Windows, GraphPad Software, La Jolla California USA, www.graphpad.com).

The comparative analysis of fertilization and hatching rates in crosses between CT females X CT males, CT females X WT males, and WT females X CT males (Fig. 5A) showed no statistical differences for those parameters (Fig. 5B). Although the rate of abnormal fish also showed no statistical differences (*p* = 0.052), the average values in the progeny derived from CT females X WT males were almost twice as high compared to other crosses (Fig. 5B).

### Comparison of survival, growth, and thermal tolerance of F1 juveniles

Survival rates of progeny from all WT groups were higher values than respective CT groups (Fig. 5C), except for WT1. Regarding growth parameters, both standard length and body weight values were similar or significantly higher (*p* ≤ 0.05) in WT in relation CT groups (Fig. 5C); in the trial 3, which recorded the highest average temperature (19.2 ± 1.29 ºC), both WT5 and WT6, showed higher body weight values than respective CT groups (*p* ≤ 0.05). In the upper temperature tolerance test at 28 ºC, F1 sub-adults and adults from WT group presented a significant superior effective time (ET; the time when fish started to lose the capacity to maintain the equilibrium) (*p* < 0.001 and *p* < 0.05, respectively), as demonstrated by the comparison between the survival curves (Fig. 5D). The LD_50_ values were 71.4% and 54.5% higher in WT group for sub-adult and adults, respectively.

## Discussion

In this study, we demonstrated that exposure to warm temperature in male juveniles affects negatively not only survival, growth, and fecundity, but also the motility of spermatozoa and body formation in the respective progeny. Despite these impacts, the progeny produced by these males showed remarkably high survival and growth rates when exposed to warm temperature compared to the control group, which supports an increased thermal tolerance after a single generation.

Long-term exposure to temperatures above optimal ranges causes detrimental effects on growth and survival in some salmonids^18,22^. Another related impact comprises the depletion of the germ cells in juveniles and adults of many other fish species^11,12,23,24^. In our experiment, heat treatment at juvenile stage did not affect reproductive parameters in females, which was not the case of males. Those fishes showed quiescent gonads that persisted for two consecutive reproductive seasons, and reduced testes, impacts that were not observed in any fish from CT group and, to the best of our knowledge, in any teleost. Since the quiescent gonads possess germ cells, we can consider these fish as infertile, but not sterile. A deep analysis on the regulatory mechanisms is required to determine how this state is controlled, if these fish still possess the capacity to resume spermatogenesis, or if steroid hormones could be used to overcome this “quiescent” condition. The significant decrease in milt volume is likely due to spermatogonial apoptosis^11,12,24,25^, because thermal treatment was performed at juvenile stage when the immature testes are composed mainly by undifferentiated spermatogonia. Regarding the difference in heat sensitivity between females and males, similar responses have been reported in teleost and mammals^12,26,27^, supporting the idea that spermatogonia possesses higher heat-sensitivity than the oogonia.

Another intriguing effect of heat treatment was observed on motility parameters of spermatozoa, whereby WT group did not show any motility after 20 s (25 and 30 s). In addition, WT spermatozoa also had higher sperm velocity and lower wobble compared to CT group at 20 s. Such patterns could be associated with high energy consumption by WT spermatozoa at the initial 5 and 10 s, based on average motility which was about 19 to 26% higher, respectively. Thus, after 20 s, WT spermatozoa would have lower available energy for motility. Reduced sperm motility after chronic heat treatment has been reported in teleosts and mammals^28,29^. In these cases, while thermal exposure encompassed mature adult males in which the spermatogenesis is under course, in our experiment, heat treatment was performed only during the pre-pubertal period. Therefore, the effects of temperature on spermatogonia persisted for about 18 months up to spermatozoa differentiation, affecting motility parameters, which suggest that warm temperature can permanently compromise the germ cells; in mammals, motility parameters were restored after a recovery period^28^. Those constitutive impacts observed in this study could be related to the particular thermal conditions during pre-pubertal developmental stage, which is characterized by high mitotic proliferation of spermatogonia^30^.

The higher proportion of deformities in progeny from WT males could also be related to this timing, as dividing cells are more prone to errors in DNA under stress conditions^31^. Thus, spermatogonia might have acquired mutations that would be deleterious for proper development of embryos. In this regard, the analysis of sperm morphology revealed that the WT group had a higher percentage of abnormal gametes when compared to the CT group. Although we do not have evidence that deformed embryos were indeed generated by those abnormal spermatozoa, studies in mice have suggested that certain types of morphologically abnormal sperm with defects in head formation are associated with a higher frequency of chromosomal mutations^32,33^. Thus, the abnormal sperm morphology due to deleterious mutations in DNA of spermatogonia could account for higher embryo malformations in WT male-derived progeny.

In spite of many negative effects on reproductive parameters of F0 males, the juveniles of the respective progeny (F1) presented remarkably superior survival and growth performances when exposed to the same conditions as those of parental fish. Moreover, sub-adults and adult progeny showed longer effective time under sublethal temperature, supporting improved thermotolerance of the F1 generation. These data may imply that warm temperature may have selected the F0 fish with genotypes that confer better thermal tolerance (WT group showed higher mortality), similarly to the reports of thermo-tolerant rainbow trout generated after several generations, as described both in the wild^34,35^ and captivity^36^. Apart from the selection at individual level, each spermatogonia from the same gonad could respond to the same stress in a different way due to genetic differences caused by temperature-induced mutations during mitotic divisions. Differences in testicular microenvironment could also induce variations in terms of epigenetics that together with genetic variation would be implicated in a selection of “stress-tolerant spermatogonia”. Thus, according to this reasoning, beneficial mutations may have been inserted into spermatogonial genome or, considering the fast acquisition of thermal tolerance, epigenetic modifications (methylation or non-coding RNAs) may have been “imprinted” onto spermatogonia in response to thermal stress and those “signatures” would be carried through the F1 progeny^37^.

The survival rates in the first trial did not differ between WT and CT males, which was affected by an outbreak of ichthyophthiriasis (a protozoosis caused by *Ichthyophthirius multifiliis*). However, in the second and third trials, WT males presented survival rates above 87% and superior growth rates compared to CT group, suggesting that chronic thermal exposure during juvenile stage may cause long-term effects on spermatozoa and on respective progeny. As mentioned previously, whether such tolerance is brought about by simple selection of genotypes with high tolerance by epigenetic modifications in the germ cell genome or transcriptome has to be further explored^38^, but regardless of the mechanism, our results open a new perspective on the capacity of organisms to overcome long-term stressful conditions within few generations. Although the final quantity of gametes and viable progeny can be severely reduced upon thermal stress, the remaining ones are able to acquire genotypes or epigenotypes that confer higher thermal tolerance into existing populations or even to replace completely these populations, thus establishing new populations^39^.

In conclusion, this study showed that warm temperature exposure in juveniles causes deleterious effects on germ cells that persist even in adults by affecting gamete production in males. Apart from germ cell degeneration, germ cell quiescence or functional sterility has to be considered as another new impact of thermal stress in fish with impairment on reproductive capacity. Nevertheless, these negative effects may be counteracted by improved survival and growth performances of progeny at warm temperature, suggesting a clear tradeoff between parental fecundity and offspring thermal resistance. These results have implications on farmed fish species, since this strategy can be used to improve tolerance to warm or high temperatures in a shorter time frame. A practical application would consist of exposing juvenile males to thermal stress, rearing them until sexual maturity, select those with lower fecundity, and use for crossing with non-exposed females for producing improved strains. From the ecological standpoint, the exposure to thermal conditions similar to those of this study that do not compromise totally the reproductive capacity in males could be harmful for impacting recruitment in populations and for generating individuals with deleterious mutations that would impair growth or reproductive fitness. But, on the other hand, if the remaining fish with acquired thermal tolerance meet conditions to reproduce and generate viable offspring, this process can be extremely beneficial to increase their adaptive capacity to cope with chronic thermal stress associated with global climate change^40–43^.

## Materials and methods

All experiments were conducted following the protocols approved by the Sao Paulo Fisheries Institute under the CEEAIP 07/2018. The procedures for the care and use of experimental fish were also approved by the institution’s committee under the same CEEAIP 07/2018. This study was carried out in compliance with the ARRIVE guidelines.

### Rainbow trout rearing conditions

This study was conducted at the governmental research hatchery Salmonid Experimental Station (SES) and in a commercial trout farm, respectively located at neighboring Campos do Jordão and Pindamonhangaba municipalities, Sao Paulo State, Brazil with a distance of 10.7 km between sites. These two sites were chosen with the aim to provide a similar water source and at conditions that approximate to those of natural environments. The hatchery (control water) is supplied by a stream running at the top of the Mantiqueira mountain at 1520 m above mean sea level (AMSL), while the farm (warm water) is supplied by a stream descending abruptly to the base of that mountain at 715 m (AMSL); both streams run through rainforest on governmental protected areas.

F0 fish used in this experiment were produced and maintained at SES until the beginning of the experiment. Six-month-old *parr* stage juveniles (body weight 22.62 ± 8.94 g; standard length 10.08 ± 1.38 cm; mean ± SD) were divided in two groups (100 per group), whereby the group transferred to the trout farm corresponded to warm temperature (WT) group (19.22 ± 1.65 °C; mean ± SD) and the one maintained at SES was the control temperature (CT) group (14.74 ± 1.63 °C; mean ± SD). In each locality, fish were subdivided into two 250 l tanks (50 per tank); the number of fish per group was chosen considering the sampling during the thermal exposure experiment for histological analysis and the mating experiments between CT and WT adults from both sexes (see below). Dissolved oxygen (DO) levels were measured weekly during the experimental period using a digital oximeter in the trout farm (8.2 ± 0.29 mg/L; mean ± SD) and at SES (8.2 ± 0.24 mg/L; mean ± SD); DO was also measured in the challenge experiment (see below). The decision on performing this study in juveniles instead of larvae or adults was based on the higher likelihood of warm or high temperatures exposure at this stage, which coincides with summer, as wild rainbow trout is born in headwaters and generally migrate downward to the sea during this season. Juveniles from both experimental groups were reared in 0.25 m^3^ round tanks for 90 days with constant water flow under natural photoperiod conditions. After this period, all animals were individually tagged (30 in WT and 36 in CT group) and transferred to a single 2 m^3^ round tank at SES for an additional period of 15 months, when most of fish reached sexual maturity. Gonad samples were collected during juvenile and adult stages for histological analyses (see scheme in Fig. 1). Animals were fed commercial diet (45% protein) twice a day ad libitum during the entire experiment.

### Histological analysis of juvenile and adult gonads

Both juvenile and adult fish were sacrificed with an overdose of benzocaine (0.2 g/L). Gonad samples were dissected, fixed in Bouinʼs solution overnight at room temperature, and stored in ethanol 70% until further processing. Samples were dehydrated in ascending ethanol series, embedded in paraffin, sectioned transversally at 5 µm thickness, and stained with hematoxylin–eosin (HE). For immunohistochemistry analysis sections were deparaffined, re-hydrated, and submitted to antigen retrieval with citrate buffer (pH 6.0) in microwave for 10 min. Endogenous peroxidase activity was blocked using 3% H_2_O_2_ solution in 0.1 M PBS at room temperature for 30 min. Sections were rinsed in 0.1 M PBS and blocked with horse serum from ImmPRESS Universal reagent (Vector Laboratories). Sections were incubated with the primary antibody #189, specific for undifferentiated spermatogonia (1:500 dilution)^44^, for 16 h at 4 °C, rinsed in 0.1 M PBS, and incubated with ImmPRESS Universal secondary antibody (Vector Laboratories) for 30 min at RT. Then, sections were rinsed in 0.1 M PBS, incubated with ImmPACT DAB HRP substrate (Vector Laboratories), counter-stained with hematoxylin and mounted.

### Analysis of growth and reproductive parameters in two-year-old fish

Total body weight (BW; g) and standard length (SL; cm) were measured for each fish. The condition factor K was calculated as K = 100 × BW/SL^3^ and the gonad weight (GW; g) was also measured for some fish to calculate the gonad-somatic index (GSI = GW/BW × 100). Reproductive parameters screened weekly by gentle abdominal pressure for ovulated females and spermiating males. Fish were anesthetized in benzocaine solution and the maximum quantity/volume of gametes were collected. This process was repeated every week only for males and for this reason the milt volume measurement was performed more than once in some fish, with the highest values considered as the milt volume for each male. Spermatozoa concentration was measured in a Neubauer chamber. For females, the total weight of ovulated oocytes and mean oocyte weight were counted. Those parameters were used to calculate the relative fecundity, which in females was presented as the oocyte number per gram of fish whereas in males it was presented as the milt volume in mL per g of fish.

The milt was diluted in buffered formaldehyde in the proportion of 1:1000 (milt: diluent solution) for spermatozoa morphological analysis. Samples were stained with Rose Bengal dye (3%) and analyzed under the microscope^45^. A total of 200 spermatozoa per male (n = 6 for each group in duplicate) were randomly selected and scored as "normal" or "abnormal", based on ovoid or triangular head shape, respectively^46^.

### Computer analysis of sperm motility

The activation process for fresh milt was performed using 1 μL of milt and 400 μL of 0.01% NaHCO_3_ (25 °C, 0.0 mOsm·kg^−1^), as activator solution. Six spermiating-males for each of WT and CT groups were randomly selected. Images and videos were captured as described in previous studies^47–49^ and processed following the description of the components required for CASA application through a free software^47^, with settings adapted from Lahnsteiner et al.^50^. Spermatozoa that presented curvilinear velocity (VCL), average path velocity (VAP), velocity in straight line (VSL), above 20, 10, and 3 µm s^−1^, respectively, were considered as motile. Besides those parameters the motility rate (MOT), straightness (STR), wobble (WOB), and progression (PROG) were also measured. Those analyses were performed for 1 s (100 images) at different post-activation times (5, 10, 15, 20, 25, and 30 s), with three videos for each male.

### Evaluation of fertilization, hatching, and abnormality rates in progeny

In the analysis using 2 year-old-fish, a total of 12 WT males were crossed with 13 CT females and conversely 6 WT females were crossed with 11 CT males (see scheme in Fig. 5A). For those crosses, 27 g of oocytes were inseminated with 2 mL of milt. In the following year, three-year-old males from WT (n = 8) and CT (n = 12) were crossed with eggs from a single CT female, in order to evaluate whether the effects observed in progeny from two-year-old-fish were restricted to fish from that age. Spermatozoa were activated using a sodium bicarbonate solution (0.01%). Eggs were then hydrated with rearing water for 20 min. and incubated at 11 °C in UV-treated water under constant flow, following conditions described in a previous study^51^. The percentage of fertilization at eyed-egg stage, hatching rate, and abnormality in eleuteroembryos were quantified for each cross. Embryos with deformities such as twisted body or swimming with circular movements were considered as abnormal.

### Survival and growth performance of WT males-derived progeny

The survival and growth performances of F1 progeny derived from WT males were compared with those from CT males. Milt from 2 WT males and 2 CT males was used to artificially inseminate the same pool of oocytes for each trial. Egg incubation and larvae rearing followed the same procedure described in the previous section. For challenge experiments under warm temperature, 300 fingerlings were randomly selected per cross. This procedure was repeated in the second trial and third trial, with 2 WT and 2 CT males each. Fish were transferred to the same commercial trout farm at Pindamonhangaba and maintained for 3 months at the same rearing conditions used for thermal treatment in broodstock fish. The temperatures and dissolved oxygen in the first, second, and third trials were 17.92 ± 1.57 °C, 7.9 ± 0.3 mg/L, 18.74 ± 1.59 °C, 8.0 ± 0.25 mg/L, and 19.20 ± 1.29 °C, 7.9 ± 0.4 mg/L (mean ± SD) at the same rearing conditions as those of WT F0 males.

### Analysis of upper temperature tolerance

Upper temperature tolerance was compared between CT and WT groups using 1-year-old sexually immature fish (n = 32 per group; BW ± SD = 305.8 ± 48.9 g and 295.5 ± 39.3 g, respectively) and 2 year-old maturing females (n = 33 per group; BW ± SD = 952 ± 184.8 g and 801.3 ± 295.1 g, respectively) from F1 generation. Fish were transferred into the temperature trial tank 24 h prior to the beginning of the experiment for acclimation. Temperature was raised from ambient (16–17 °C) to upper lethal temperature of 28 °C, following the methodology described by Jackson et al*.* 1998^52^. Dissolved oxygen levels were maintained at a minimum of 8.0 mg/L. The time when fish started to lose the capacity to maintain the equilibrium was recorded for all individuals and considered as the ‘effective time’ (ET) in the zone of thermal resistance. LT_50_ (median lethal temperature) was calculated for each curve.

### Sex genotyping by sdY amplification

Total genomic DNA was extracted from the caudal fin of all 2 year-old F0 fish (n = 61) using saline buffer method^53^ and used for PCR amplification of *sdY* gene (sexually dimorphic on the Y chromosome). Primers and amplification conditions followed^54^ and PCR products were electrophoresed in 1% agarose gel stained with Ethidium bromide.

### Statistical analysis

Data were analyzed in GraphPad Prism (version 5.02 for Windows, GraphPad Software, La Jolla California USA, www.graphpad.com) using the analysis of variance (One-way ANOVA), followed by Tukey’s multiple comparisons test. All data were presented as mean ± S.E.M. The GSI in juveniles and spermatozoa parameters were compared using Student’s t-test. The comparison of effective time was performed by a survival test, with the curves compared based on Gehan-Breslow-Wilcoxon test. Differences were considered as significant for *p* ≤ 0.05.

## Supplementary Information

Supplementary Information.
