# Supplementary material for: Early warming stress on rainbow trout juveniles impairs male reproduction but contrastingly elicits intergenerational thermotolerance
Source: Sci Rep. 2021 Aug 23;11:17053. doi: 10.1038/s41598-021-96514-1 (PMC8382822; doi:10.1038/s41598-021-96514-1)
Supplement: Supplementary file 1 — Supplementary Information. [file 41598_2021_96514_MOESM1_ESM.pdf]

Early warming stress on rainbow trout juveniles impairs testicular development and sperm quality but contrastingly elicits intergenerational thermotolerance

Arno Juliano Butzge[1], Tulio Teruo Yoshinaga[2], Omar David Moreno Acosta[3], Juan Ignacio Fernandino[3], Eduardo Antônio Sanches[4], Yara Aiko Tabata[5], Claudio de Oliveira[1], Neuza Sumico Takahashi[6], Ricardo Shohei Hattori[5*]

Supplementary material

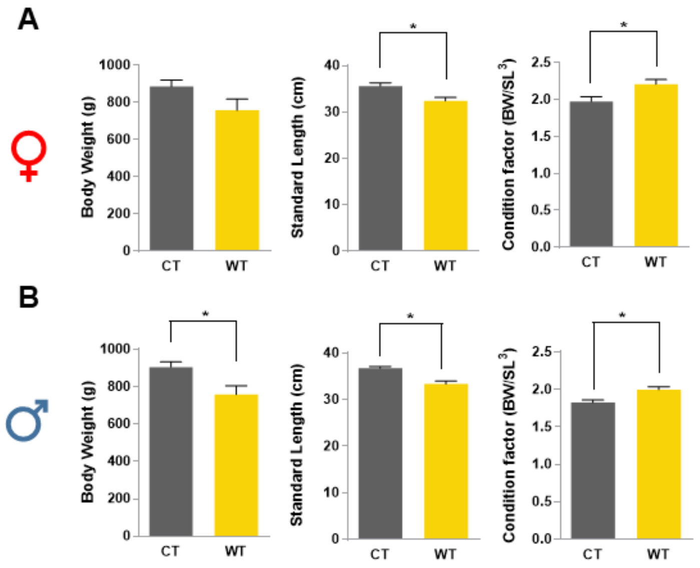

Supplementary figure 1. Growth parameters from F0 adult animals. WT females presented lower standard length and higher K condition factor compared to CT group (**A**). WT males presented lower values for both weight and standard length, and hence higher K condition factor values than control group (**B**).

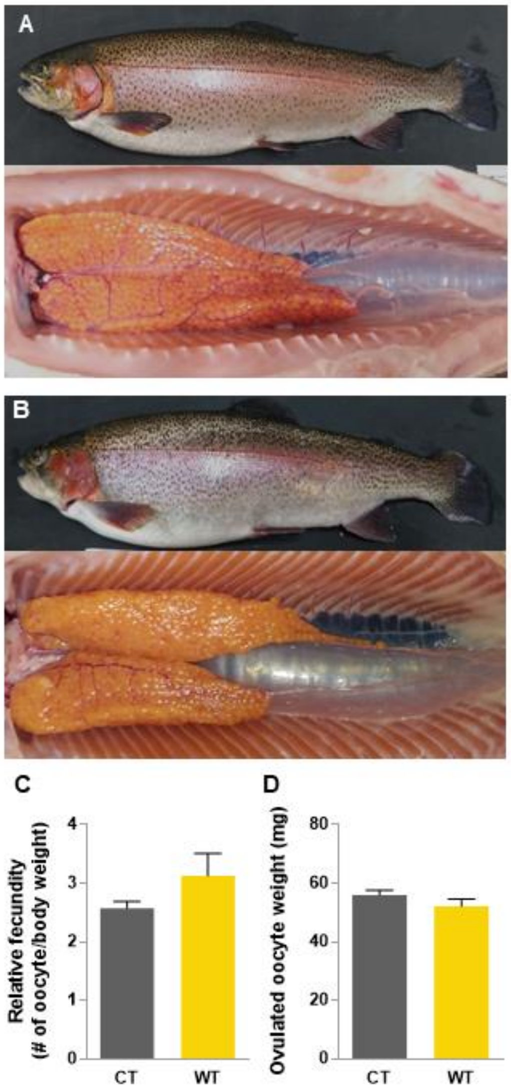

Supplementary figure 2. External body appearance, morphology of gonads, and fecundity in control (**CT**) and warm temperature (**WT**) females. Body and ovary morphologies of rainbow trout females from CT (**A**) and WT (**B**) groups. Comparison of (**C**) relative fecundity and (**D**) oocyte size.
